# Consequences of Gift Giving in Online Health Communities on Physician Service Quality: Empirical Text Mining Study

**DOI:** 10.2196/18569

**Published:** 2020-07-30

**Authors:** Li Peng, Yanan Wang, Jing Chen

**Affiliations:** 1 Tongji Hospital, Tongji Medical College, Huazhong University of Science and Technology Wuhan, Hubei China; 2 School of Medicine and Health Management, Tongji Medical College, Huazhong University of Science and Technology Wuhan, Hubei China; 3 Department of Radiology, Central South University Xiangya School of Medicine Affiliated Haikou Hospital Haikou China

**Keywords:** online health community, gift giving, affective/instrumental gifts, service quality, bedside manner, physicians, physician-patient relationship

## Abstract

**Background:**

Gift giving, which has been a heavily debated topic in health care for many years, is considered as a way of expressing gratitude and to be beneficial for the physician-patient relationship within a reasonable range. However, not much work has been done to examine the influence of gift giving on physicians’ service quality, especially in the online health care environment.

**Objective:**

This study addressed the consequences of gift giving by mining and analyzing the dynamic physician-patient interaction processes in an online health community. Specifically, gift types (affective or instrumental) based on the motivations and physician-patient tie strength were carefully considered to account for differences in physicians’ service quality.

**Methods:**

The dynamic interaction processes (involving 3154 gifts) between 267 physicians and 14,187 patients from a well-known online health community in China (haodf.com) were analyzed to obtain empirical results.

**Results:**

Our results reveal that patient gift giving inspires physicians to improve their service quality as measured by physicians’ more detailed responses and improved bedside manner, and the degree of influence varied according to the strength of the physician-patient tie. Moreover, affective gifts and instrumental gifts had different effects in improving physicians’ service quality online.

**Conclusions:**

This study is among the first to explore gift giving in online health communities providing both important theoretical and practical contributions. All of our results suggest that gift giving online is of great significance to promoting effective physician-patient communication and is conducive to the relief of physician-patient conflicts.

## Introduction

### Background

Online health communities have recently emerged as an important channel for seeking medical information and physicians’ help [1], improving the physician-patient relationship [2], and raising the level of public health [3]. The Pew Research Center reported that 59% of American adults had searched for health information online [4]. An increasing number of scholars have been paying specific attention to patient [5,6] or physician [7-9] behaviors in online health communities. However, little is known about how patient behaviors influence physician behaviors.

Patient behaviors online can be mainly divided into purchase and review behaviors. The former has already been investigated in a large number of studies. With respect to review behaviors, positive behaviors were shown to account for almost 99% of all reviews (calculated based on data collected on Haodf.com [10]), which might be due to the unequal relationships between patients and physicians. Among the various ways of expressing gratitude, gift giving is popular among patients, although this behavior has been widely questioned by the public [11]. One cause of these debates is that the possible impact of gift giving on the service quality of physicians remains unclear. Both academia and industries have raised the question as to whether patients buy gifts for physicians in fear of receiving poor treatment rather than as a gesture of expressing gratitude [12]. To address these questions, we have been exploring patients’ motivations of gift giving as well as the impact of gift giving on physician behavior (ie, service quality).

Within a reasonable rage, gift giving has benefits for emotional expression and relationship building in interpersonal communication [13] and can be considered to be a benefit for the physician-patient relationship. From the patient perspective, on the one hand, gifts can be used to express gratitude. On the other hand, gift giving helps to reassure patients that physicians will be more likely to fulfill their responsibilities after receiving gifts. From the physician perspective, on the one hand, gifts from patients make them feel respected and recognized for their efforts. On the other hand, gifts may inspire them to improve service quality.

Despite recognition of the many advantages of gift giving for both patients and physicians, these effects should also be empirically examined. To the best of our knowledge, only one study has investigated the relationships between gift giving and physicians’ service delivery to date [14]. However, the influence according to gift types distinguished by motivations has not been given full consideration. Moreover, the influences of gift giving on physicians may demonstrate time effects, which have also not been analyzed. In the previous study, service quality was measured by the physicians’ response speed to patients’ question. Response speed, as a dimension of service quality, will ultimately influence patients’ perceived service quality. However, we believe that the quality of the reply content to the question may be more important and highly valued by patients.

To extend existing studies, we systematically analyzed the motivations and effects of gift giving in online health care. First, gifts were classified into affective gifts and instrumental gifts based on different motives [15]. All gifts that physicians received were judged and categorized based on the complete interaction process (including gift giving) between physicians and patients. The different effects of affective and instrumental gifts on physicians’ service quality were further examined. Second, the degree of intimacy of the physician-patient relationship (ie, the tie strength) may influence the effects of gift exchange [16] and was also considered in our study. Third, the time effects of gift giving were comprehensively analyzed to obtain conclusions that can offer more practical guidance to patients. Fourth, the level of detail and emotional support of physicians’ replies were used to measure service quality. Therefore, the specific research questions in this study were as follows: (1) Does gift giving influence physicians’ service quality in online health communities? (2) Are the effects of different types of gifts (affective and instrumental gifts) on physicians’ service quality consistent? (3) How does the physician-patient tie strength moderate the relationships between gift giving and service quality? and (4) Does gift giving show time effects on service quality?

To address these questions, we adopted a quantitative approach and examined the effect of gift giving for the whole interaction process on physicians’ service quality by analyzing longitudinal data gathered from a popular online health community in China, Haodf.com [10].

### Gift Theory

Gift exchange plays a vital role in social interactions [17]. Gift theory, which involves the norm of reciprocity and explains why gift givers frequently receive return gifts, provides a suitable theoretical basis for this study.

The principle of reciprocity, which is considered as a benefit for individuals participating in social exchange, is often defined as a set of socially accepted trading rules in which one party provides resources to the other and obliges the other to return the favor [18]. The reciprocity principle is the internal cause of continuous communication between people since people will always repay each other owing to the nature of mutual indebtedness within the principle [19]. Moreover, referring to the partition manner of gifts in sociology, we divide gifts into affective and instrumental gifts based on different motives. Affective and instrumental gifts are defined as having “emotional expression” and “utilitarian purpose,” respectively. However, both affective and instrumental elements, with different proportions, rarely exist in the process of gift exchange simultaneously. Hence, the gift type in our study was defined as the major element in a given exchange.

### Professional Identity

Professional identity is defined as how one perceives the goals, social values, and other factors of their profession, and how this is communicated to others [20]. Relevant literature has paid more attention to the professional identity of physicians, nurses, and teachers; there are diverse definitions of professional identity in different fields [21,22], although this identity is of relevance for each worker. Only when one recognizes the occupation they are engaged in can they become more devoted to the work, exert their full potential, and realize their value in the course of the work. Studies concerning professional identity have revealed that high professional identity can be viewed as a unique way of shaping a good professional image, whereas the absence of such identity may drive one to leave the field [23]. Furthermore, professional identity can be enhanced through recognition and encouragement from others [24].

### Tie Strength Theory

Following prior studies, tie strength is defined as the level of intimacy and interaction between individuals. Granovetter [25] conducted a comprehensive study to define and classify tie strength, which was categorized according to weak and strong ties defined by “unfamiliar interpersonal relations” and “familiar interpersonal relations,” respectively. Thus, strong ties reflect closer relationships and more frequent interactions compared to weak ties [26]. Therefore, in the context of online health communities, tie strength can be considered to consist of two dimensions, intimacy and interaction, which are measured by the cumulative number of gifts and interaction times, respectively. Strong ties can provide material and reliable support, whereas weak ties, along with multiple information, cannot [27]. Hence, the strength of ties determines the quality of the information available and the likelihood that individuals will achieve their goals [28].

We therefore framed our study based on these perspectives to examine how tie strength influences the effect of giving gifts in online health communities. The judgment on gift types and the subsequent attitude about gift giving will both be influenced by the relationships between receivers and givers [29]. Specifically, gift giving is more likely to be accepted when the two sides are in a close relationship such as family, romantic partnership, friendship, and even geographical proximity. However, the receivers can easily suspect the motives of the gift, which will then be refused when the receivers and givers are strangers. Hence, we surmise that tie strength will not only moderate the impact of gift giving on physicians’ service quality but will also influence the physicians’ judgment of gift types.

### Research Model and Hypotheses

**Figure 1:**
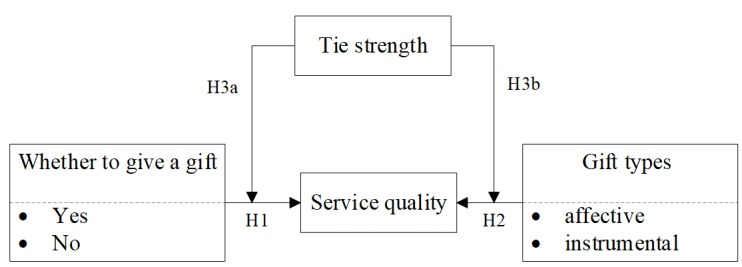
Conceptual model.

Figure 1 shows the proposed research model based on the above theoretical background. To understand the relationships among several constructs, we proposed and tested several hypotheses.

Gift theory states that receivers will have the moral norm and obligation of returning the favor because of the reciprocity norm [30]. Online virtual gifts provide physicians with economic and honorary utility. Therefore, physicians always face the obligation and motivation to reciprocate patients after receiving a gift. Considering the online health care environment, we believe that physicians may improve their service quality to patients after receiving a gift, leading to the following hypothesis (H1): gifts help improve physicians’ service quality in online health communities.

Previous studies based on the offline environment found that gift exchange offers a way to connect socially [31]. However, gifts provided at different times and different communication stages may have different effects. Even if we consider that all gifts are beneficial to the improvement of physicians’ service quality, gifts with different purposes may have different effects. Kirchler and Palan [32] tested the value of unconditional nonmonetary gifts as a way to improve health worker performance in a low-income country health setting, and found that unconditional nonmonetary gifts enhanced performance by 20% over 6 weeks compared to conditional nonmonetary gifts.

Affective gifts are used to express the givers’ appreciation, regardless of the possibility of return, whereas instrumental gifts are often given for a utilitarian purpose. Compared with instrumental gifts, affective gifts can provide not only economic benefits and improvement of online reputation [33] but also encouragement and recognition from patients, which can in turn enhance the professional identity of physicians and then inspire them to improve their service quality. Based on these perspectives, we developed our second hypothesis (H2) as follows: compared with instrumental gifts, affective gifts will be more effective in improving physicians’ service quality.

Tie strength affects the quality of the information available and the likelihood that individuals will achieve their goals; a strong tie helps to obtain a gift that is more expensive and valuable [27]. When a patient and a physician know each other well, affectivity and compulsoriness will play major roles in their interaction process [26]. In such a situation, physicians always serve patients conscientiously. Therefore, a strong tie may weaken the effect of gift giving on physicians’ service quality, leading us to establish the following hypothesis (H3a): tie strength negatively moderates the relationships between patients’ gift giving and physicians’ service quality.

The lower the degree of intimacy between givers and receivers, the more likely people regard gifts with utilitarian features [29]. Therefore, tie strength may affect the receiver’s judgment on the motivations of gifts. Specifically, strong ties between physicians and patients decrease the feelings of physicians on the differences between affective and instrumental characteristics. A strong tie between a physician and patient will mitigate the effects of gift types on the physician’s behavior. Based on these perspectives, we developed the following hypothesis (H3b): tie strength mitigates the difference of the effect of gift type on physicians’ service quality.

## Methods

### Research Context

**Figure 2:**
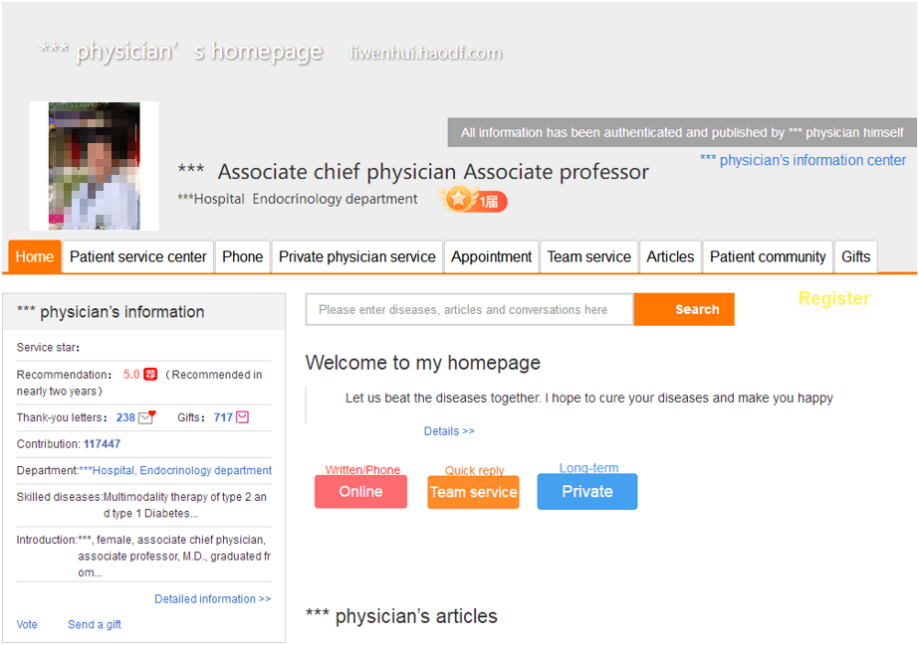
Physician’s homepage.

**Figure 3:**
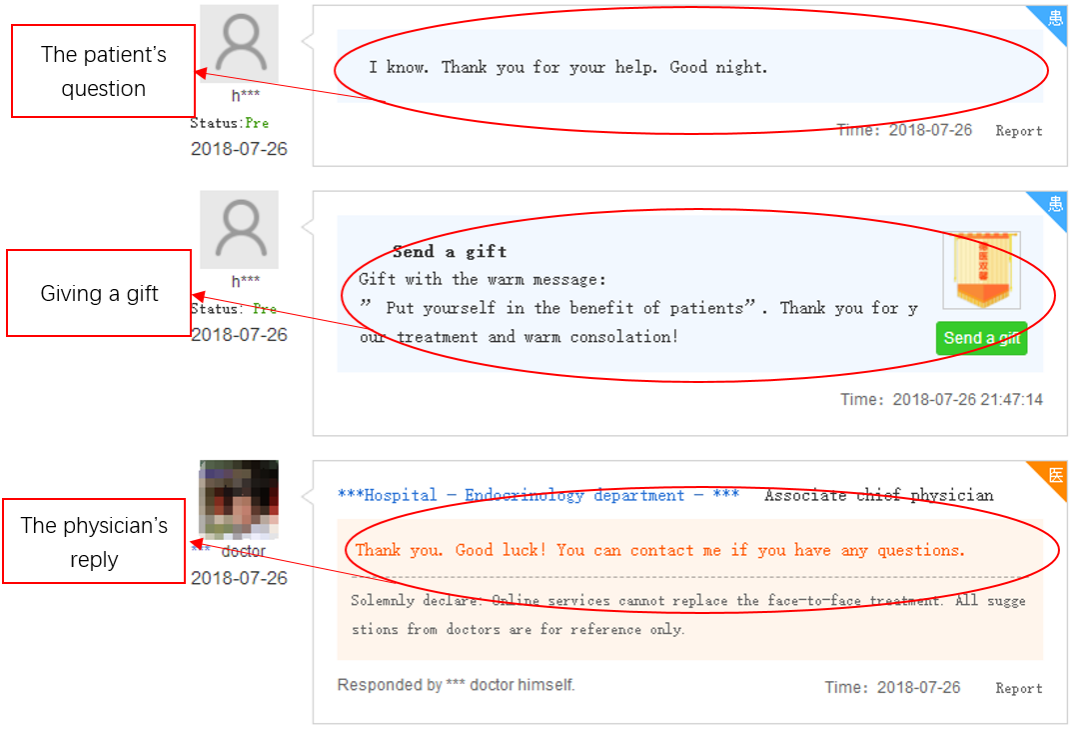
Physician-patient interaction process.

To test the research hypotheses, we collected data from one of the most popular online health communities, Haodf.com [10], which was established in 2006 in China. More than 490,000 physicians from 7500 hospitals are currently working on the site. Haodf.com creates a homepage for each physician (see Figure 2; all screenshots of web pages were translated to English for clarity). For each physician, the entire interaction process with all patients is archived on the site. The interaction process includes the communicator (physician or patient), time, and content (physician-patient dialog or patient gift giving) for each interaction. An example of the interaction process is shown in Figure 3. Data collection was facilitated since most of the physician-patient interaction processes on Haodf.com are public, except for a few processes that are encrypted for privacy protection.

### Sample and Data Collection

To reduce the influence of disease types, we only included physicians who treat patients with diabetes as our sample. We developed a crawler to automatically download information, including physicians’ information and each physician-patient interaction content, on Haodf.com. We collected the data on March 25, 2018, and the entire process was conducted for 1 week. After downloading, cleaning up, and matching information processes, 217,458 interactions (*X_1_*) between 14,187 patients and 267 physicians were included in our model.

**Figure 4:**
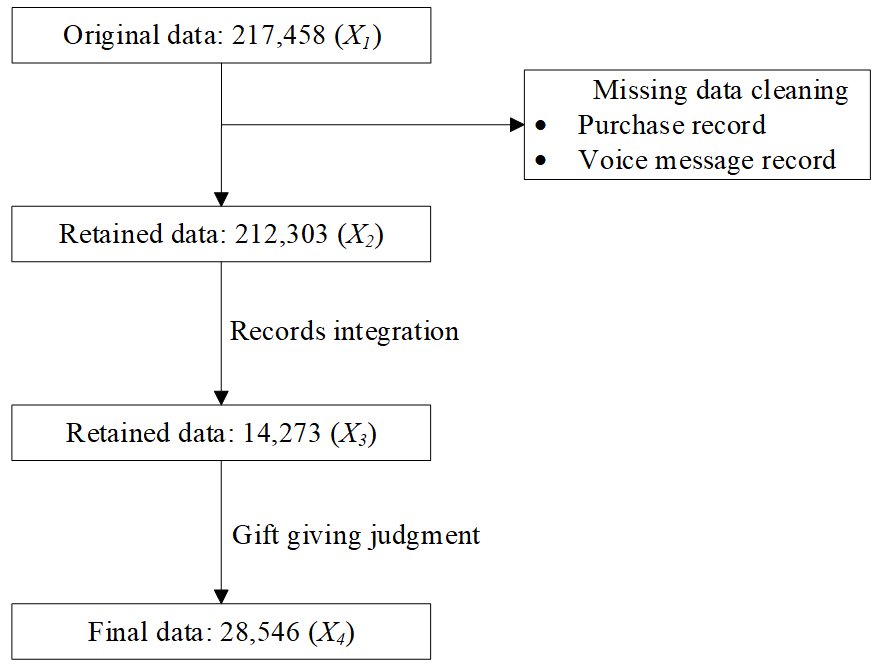
Data cleaning and processing.

**Figure 5:**
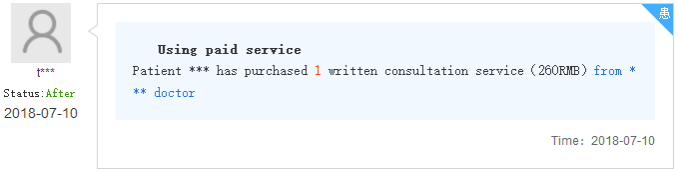
Patient’s purchase process.

**Figure 6:**
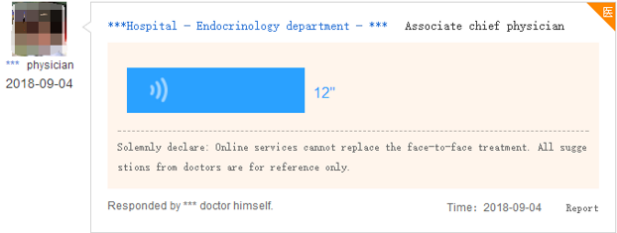
Physician’s reply by voice message.

The data cleaning and processing (see Figure 4) were broken down into the following steps: (1) cleaning missing data, in which the records for patients’ purchase behaviors (see example in Figure 5) and physicians’ replies by voice messages (see example in Figure 6) were deleted, resulting in 212,303 records (*X_2_*) retained; (2) records integration (*X_3_*) from each physician-patient interaction process per day to ensure communication continuity; and (3) gift giving judgment, in which each record in *X_3_* was divided into two records based on the first gift giving behavior, namely prior to and after gift giving. Finally, 28,546 records (*X_4_*) were included in our model.

### Judgment of Gift Types and Tie Strength

Judgment of gift types and tie strength was based on text mining.

#### Manual Coding for Gift Types

**Figure 7:**
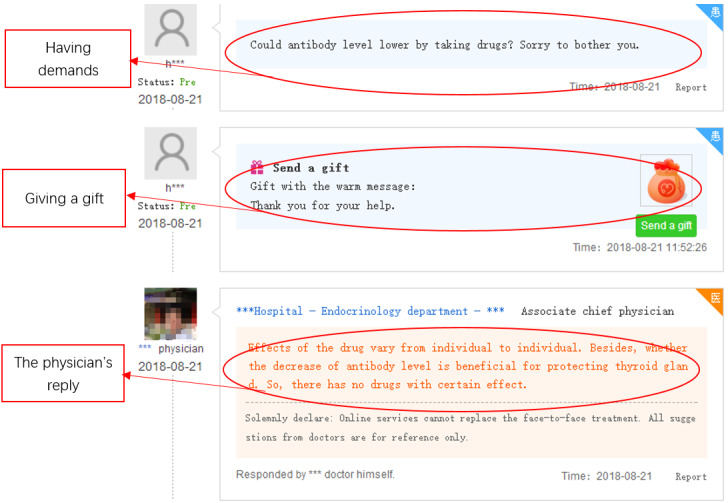
Instrumental gift example.

**Figure 8:**
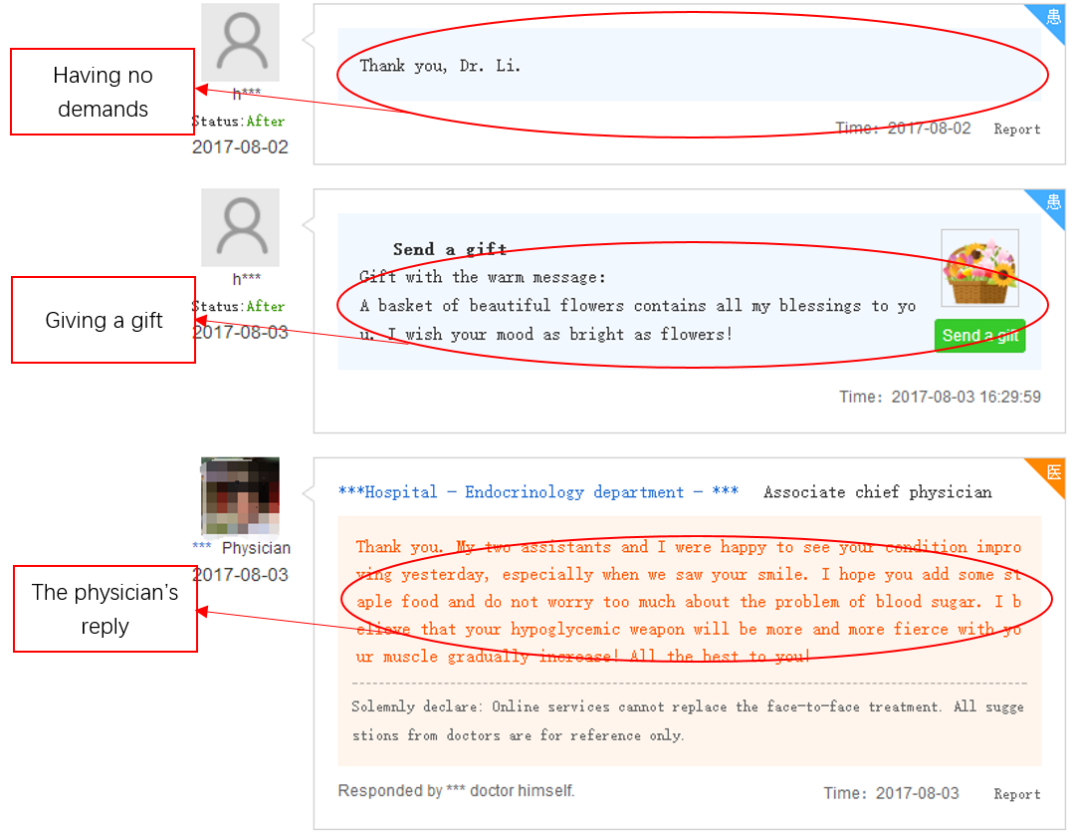
Affective gift example.

Gift types were judged according to whether gift giving occurred in conjunction with patients’ questions. An instrumental gift is one that is purposefully given to physicians (eg, with the goal of obtaining better service). Therefore, gifts that are given close to a patient’s questions were considered to be instrumental gifts (see Figure 7 for an example); otherwise, the gift was considered to be affective (see Figure 8 for an example). With respect to the judgment of whether the dialog involved a question, two assistants with a research background in medical informatics were trained to recognize patients’ questions based on the keywords shown in Multimedia Appendix 1. One hundred whole interaction processes, including 300 dialogs between patients and physicians, were chosen at random and assigned to be judged. The two assistants coded the contents of the 300 dialogs independently, and consistent judgment was obtained for 296 (98.7%) dialogs. After analyzing the remaining ambiguous dialogs, consistent judgments were reached. Finally, one assistant was assigned to code the remaining contents.

#### Measurement of Tie Strength

Tie strength was measured based on the cumulative quantity of gifts and conversations. A conversation includes a question and a reply. Therefore, the tie strength will increase as the number of physician replies increases. We standardized the cumulative quantity of gifts and conversations, respectively, and tie strength was calculated according to the following formula: standardized number of gifts + standardized number of physician replies.

### Variables and Empirical Models

The empirical variables included in our model are shown in Table 1. As the dependent variable, we used the ratio of average word count for a physician to respond to the patient in dialog *i* as a measure of service quality. More words contain more information. Specifically, a long reply may cover solutions for the question from all aspects and offer a detailed explanation of each aspect. In addition, a long reply may reflect the physician’s patience and serious attitude to their patients. We also considered that a long question may lead to a long reply. Therefore, we also controlled for the word count from the patient’s question. The formula is as follows: word count ratio*_i_*=ln(average word count for physician dialog *i*)/ln(average word count for patient in dialog *i*).

**Table 1:** Variables description.

Variable		Explanation
*Ratio_WordCount*_ij_ (Dependent variable)		The ratio of average word counts for physician *j* to that of the patient in dialog *i*
**Independent variables**		
	*GiftGiving*_ij_	Dummy variable in which “1” represents that a gift has been sent and “0” represents no gift
	*GiftType*_ij_	Dummy variable in which “1” represents an instrumental gift and “0” represents an affective gift
	*TieStrength*_ij_	Summation of standardized interaction times and the standardized cumulative number of gifts
**Control variables**		
	*Phone*_j_	Dummy variable in which “1” represents that the physician provides phone consultation and “0” represents no phone consultation
	*Written*_j_	Dummy variable in which “1” represents that the physician provides written consultation and “0” represents no written consultation
	*Outpatient*_j_	Dummy variable in which “1” represents that the physician provides outpatient service appointments and “0” represents no appointment provision
	*PhonePrice*_j_	Service price for phone consultation service set by the physician
	*WrittenPrice*_j_	Service price for written consultation service set by the physician
	*Thank-you Letter*_j_	Number of thank-you letters that physician *j* received
	*No. Gifts*_j_	Number of gifts that physician *j* received
	*Contribution*_j_	Calculated by site to measure the effort of physician *j*
	*No. Patients*_j_	Number of patients that physician *j* consulted
	*Recommendation*_j_	Total number of votes for each physician from patients
	*Title*_j_	Dummy variable in which “1” represents that physician *j* is a chief physician and “0” otherwise
	*City*_j_	Economics of the city where the hospital is located to measure patients’ consumption capacity. Expressed as a dummy variable in which “1” represents that the physician works in a first-tier city and “0” otherwise
	*Level*_j_	Evaluated by the government reflecting the hospital’s ability, equipment, and technology. Expressed as a dummy variable in which “1” represents that the physician works in a “III A” level hospital and “0” otherwise

Three independent variables were included in our study: (1) whether physician *j* received gifts in dialog *i* (gift giving), (2) gift type (affective or instrumental), and (3) tie strength.

To control for heterogeneity among physicians, we collected physicians’ information online. Haodf.com provides three primary services for physicians to choose whether to provide, including written consultation, phone consultation, and an outpatient service appointment (details can be found in [33]). Three dummy variables were used to measure the three services. If a physician provided a written or phone consultation, the service price was also included in the model. The physician’s professional title (chief, associate chief, or attending physician) evaluated by the health sector was also included. The detailed explanations for other control variables are provided in Table 1.

Our analyses were conducted in two steps. In the first step, multiple linear regression was used to explore the impacts of patients’ gift giving (whether to give a gift) on physicians’ service quality. In the second step, based on the situation in which gift giving occurred, the impacts of gift types and tie strength on physicians’ service quality were examined. Therefore, only dialogs including gift giving were included in this step. Our empirical models for each step are as follows:

Step 1: Word count ratio = α*_ij_* + α*_1_* × *GiftGiving_ij_* + α*_2_* × *Control_j_* + ε*_j_*

Step 2: Word count ratio = α*′_ij_* + α*′_1_* × *GiftGiving_ij_* + α*′_2_* × *Control_j_* + ε*′_j_*

Word count ratio = α*′′_ij_* + α*′′_1_GiftGiving_ij_* + α*′′_2_* × *TieStrength_ij_* + α*′′_3_* × *GiftGiving_ij_* × *TieStrength_ij_* + α*′′_4_* × *Control_j_* + ε*′′_j_*

Word count ratio = α*′′′_ij_* + α*′′′_1_GiftType_ij_* + α*′′′_2_* × *TieStrength_ij_* + α*′′′_3_* × *GiftType_ij_* × *TieStrength_ij_* + α*′′′_4_* × *Control_j_* + ε*′′_j_*

where *i* denotes a dialog and *j* denotes a physician; α is the coefficient to be estimated. *Control_j_* represents the control variables for physician *j*, and ε*_j_* is the standard error.

## Results

### Descriptive Statistics and Correlations

The descriptive statistics and correlations for the key variables are presented in Multimedia Appendix 2. Independent variables were significantly related to the dependent variable. The correlation matrix showed no serious multicollinearity, which assured obtaining reliable results.

### Empirical Results

The multiple linear regression method was applied to examine the effect of patients’ gift giving on physicians’ service quality, and the empirical results are shown as Model 1 (adjusted *R^2^*=0.018, *F*_change_=19.774, *P*<.001) and Model 2 (adjusted *R^2^*=0.027, *F*_change_=27.171, *P*<.001). We found that the decision to give gifts positively influenced physicians’ service quality, thereby supporting hypothesis H1 (Table 2).

We then investigated the impacts of gift types and tie strength on physicians’ service quality. The results are shown in Model 3 (adjusted *R^2^*=0.031, *F*_change_=19.737, *P*<.001). Compared with instrumental gifts, affective gifts had a stronger influence on physicians’ service quality, thereby supporting hypothesis H2 (Table 2).

In Model 4 (adjusted *R^2^*=0.017, *F*_change_=11.184, *P*<.001), we examined the moderating effect of tie strength on the relationship between gift giving and physicians’ service quality. We found that the relationship between patients’ gift giving and physicians’ service quality was smaller when they had a strong tie (Table 2). In Model 5 (adjusted *R^2^*=0.035, *F*_change_=19.071, *P*<.001), a positive moderating effect of tie strength on the relationship between gift types and physicians’ service quality was identified (Table 2). The relationship between gift types and physicians’ service quality was smaller when there was a strong tie between the physician and patient, thereby supporting hypotheses H3a and H3b.

**Table 2:** Regression results on associations of gift giving and physical service quality.

Variables	Step 1, β (SE)					Step 2, β (SE)					
	Model 1	*P* value	Model 2	*P* value	Model 3		*P* value	Model 4	*P* value	Model 5	*P* value
Intercept	3.251 (0.344)	<.001	2.972 (0.344)	<.001	2.532 (-0.443)		<.001	2.775 (0.445)	<.001	2.513 (0.443)	<.001
*PhonePrice*	–0.034 (0.033)	.18	–0.017 (0.033)	.18	–0.137 (–0.044)		.008	–0.172 (0.044)	<.001	–0.143 (0.044)	.008
*WrittenPrice*	–0.040 (0.031)	.30	–0.052 (0.031)	.21	0.043 (–0.040)		.35	0.058 (0.041)	.35	0.044 (0.040)	.35
*Outpatient*	0.099 (0.035)	.009	0.111 (0.034)	<.001	0.024 (–0.045)		.42	0.024 (0.046)	.42	0.026 (0.045)	.42
*Thank-you Letter*	0.194 (0.033)	<.001	0.183 (0.033)	<.001	0.263 (0.044)		<.001	0.284 (0.044)	<.001	0.265 (0.044)	<.001
*Contribution*	–0.088 (0.060)	.31	–0.056 (0.060)	.31	0.137 (0.076)		.10	0.075 (0.077)	.31	0.137 (0.077)	.10
*No. Gifts*	–0.172 (0.030)	<.001	–0.188 (0.030)	<.001	–0.207 (0.039)		<.001	–0.156 (0.039)	<.001	–0.190 (0.039)	<.001
*No. Patients*	0.315 (0.060)	<.001	0.306 (0.059)	<.001	0.001 (0.078)		.01	0.002 (0.078)	.01	–0.018 (0.078)	<.001
*Recommendation*	–0.493 (0.076)	<.001	–0.506 (0.076)	<.001	–0.253 (0.097)		.004	–0.265 (0.098)	.004	–0.247 (0.097)	.004
*Title*	0.026 (0.035)	.54	0.020 (0.035)	.55	0.096 (0.046)		.05	0.084 (0.047)	.10	0.088 (0.046)	.10
*City*	0.083 (0.040)	.06	0.088 (0.040)	.06	-0.032 (0.053)		.57	-0.017 (0.054)	.89	-0.026 (0.053)	.55
*Level*	–0.292 (0.048)	<.001	–0.285 (0.048)	<.001	–0.268 (0.061)		<.001	–0.279 (0.062)	<.001	–0.256 (0.061)	<.001
*GiftGiving*	N/A^a^	N/A	0.300 (0.029)	<.001	N/A		N/A	N/A	N/A	N/A	N/A
*GiftType*	N/A	N/A	N/A	N/A	–0.415 (0.038)		<.001	N/A	N/A	–0.329 (0.051)	<.001
*GiftGiving* *×* *TieStrength*	N/A	N/A	N/A	N/A	N/A		N/A	–0.104 (0.025)	<.001	–0.137 (0.026)	<.001
*GiftType* *×* *TieStrength*	N/A	N/A	N/A	N/A	N/A		N/A	N/A	N/A	0.209 (0.077)	.04

^a^N/A: not applicable.

### Robustness Check

The dependent variable in our study was physicians’ service quality, which was measured by the ratio of the average word count for a physician in the patient in dialog *i*. Specifically, we divided the logarithm of the average word count for a physician by the logarithm of the average word count for the patient to calculate physicians’ service quality. Robustness was calculated by dividing the average word count for a physician by that of the patient (see Table 3). These values were consistent with the main results of the model in Table 2, which assured the good robustness of the results.

**Table 3:** Robustness check.

Variables	Step 1, β (SE)					Step 2, β (SE)					
	Model 1^a^	*P* value	Model 2^b^	*P* value	Model 3^c^		*P* value	Model 4^d^	*P* value	Model 5^e^	*P* value
*GiftGiving*	N/A^f^	N/A	1.154 (0.692)	.10	N/A		N/A	N/A	N/A	N/A	N/A
*GiftType*	N/A	N/A	N/A	N/A	–7.332 (0.828)		<.001	N/A	N/A	–5.732 (1.114)	<.001
*GiftGiving* *×* *TieStrength*	N/A	N/A	N/A	N/A	N/A		N/A	–1.707 (0.533)	.009	–2.316 (0.561)	<.001
*GiftType* *×* *TieStrength*	N/A	N/A	N/A	N/A	N/A		N/A	N/A	N/A	3.866 (1.681)	.06

^a^Adjusted *R^2^*=.014, *F*_change_=15.907, *P*<.001.

^b^Adjusted *R^2^*=0.014, *F*_change_=14.816, *P*<.001.

^c^Adjusted *R^2^*=0.027, *F*_change_=17.007, *P*<.001.

^d^Adjusted *R^2^*=0.017, *F*_change_=11.226, *P*<.001.

^e^Adjusted *R^2^*=0.029, *F*_change_=15.899, *P*<.001.

^f^N/A: not applicable.

### Posthoc Analysis

We further tested the impacts of gift giving on physicians’ bedside manner, which is also important for patients and a dimension of service quality. Text mining was used to analyze physicians’ replies and to judge their attitude. TextMind, a psychoanalytic software system developed by the Chinese Academy of Sciences for language analysis (especially for Chinese), was used for this analysis. This software has been widely used to analyze various characteristics of the text content in previous studies (eg [34]). By using TextMind, we conducted a sentimental analysis for physicians’ text content. The specific process is shown in Multimedia Appendix 3.

The average number of the physician positive sentiment words in each dialog was used to measure the physician’s bedside manner. As shown in Table 4, we found a positive impact of gift giving on physicians’ bedside manner; however, the impact was smaller when the tie strength was strong (Model 2). In addition, compared with instrumental gifts, affective gifts had a greater impact on physicians’ bedside manner (Model 3), but the gap was reduced when the tie strength was stronger (Model 5). These results are consistent with our main results, indicating that gift giving works effectively with respect to both the physicians’ reply and bedside manner.

**Table 4:** Empirical model results for physician bedside manner.

Variables	Step 1, β (SE)					Step 2, β (SE)					
	Model 1^a^	*P* value	Model 2^b^	*P* value	Model 3^c^		*P* value	Model 4^d^	*P* value	Model 5^e^	*P* value
*GiftGiving*	N/A^f^	N/A	0.342 (0.023)	<.001	N/A		N/A	N/A	N/A	N/A	N/A
*GiftType*	N/A	N/A	N/A	N/A	–0.348 (0.030)		<.001	N/A	N/A	–0.239 (0.041)	<.001
*GiftGiving* *×* *TieStrength*	N/A	N/A	N/A	N/A	N/A		N/A	–0.164 (0.024)	<.001	–0.196 (0.026)	<.001
*GiftType* *×* *TieStrength*	N/A	N/A	N/A	N/A	N/A		N/A	N/A	N/A	0.232 (0.062)	<.001

^a^Adjusted *R^2^*=0.028, *F*_change_=16.743, *P*<.001.

^b^Adjusted *R^2^*=0.064, *F*_change_=34.801, *P*<.001.

^c^Adjusted *R^2^*=0.056, *F*_change_=19.318, *P*<.001.

^d^Adjusted *R^2^*=0.078, *F*_change_=26.869, *P*<.001.

^e^Adjusted *R^2^*=0.091, *F*_change_=27.562, *P*<.001.

^f^N/A: not applicable.

## Discussion

### Principal Findings

The aim of this study was to determine the main motivations and effects of online virtual gift giving on physicians’ service quality in online health communities using text mining and econometric methods. Specifically, we evaluated the impact of whether to give gifts and gift types systematically. Moreover, tie strength was carefully considered in the model as it may influence the effects of gift giving. Further, both the physicians’ reply and bedside manner were used to measure service quality. The empirical results support all of our hypotheses.

Gift giving can enhance physicians’ service quality, which was proven in our model and is consistent with prior studies [14]. As an important tool in social interactions [17], gift exchange is beneficial for individuals to participate in social contact. A reciprocity norm exists among people and is scrupulously obeyed; that is, people always repay each other for the mutual indebtedness within the reciprocity principle [19]. For example, in return for gifts from employers, workers will be more productive [35]. Therefore, we believe that physicians will improve their service quality when they have received gifts from patients under the reciprocity principle. In addition, gifts provide psychological gratification to the physician, as gifts from patients make them feel respected and recognized for their effort, and thus inspire them to improve their service quality.

Gifts with different motivations had different effects on influencing physicians’ behaviors. We extended existing studies on gift types (eg, [15]) by investigating the consequences for giving different types of gifts, and found that although gift giving can inspire physicians to improve their quality overall, affective gifts play a more prominent role in enhancing quality compared with instrumental gifts. Our results are consistent with a previous study [32] that examined the impact of unconditional nonmonetary gifts on health worker performance in a low-income country in which unconditional nonmonetary gifts improved the performance of workers by 20% in 6 weeks compared with conditional nonmonetary gifts. Affective gifts are unconditional nonmonetary gifts, and instrumental gifts are similar to conditional nonmonetary gifts.

With respect to the moderating effects of tie strength between physicians and patients, we found that the more familiar a physician is with their patient, the role of whether to send gifts and gift type on physician service quality is decreased. Strong ties improve people’s trust and obligation. Although weak ties play an important role in information transmission, a strong tie helps to obtain something more expensive and valuable [27]. Specifically, a strong tie between a physician and their patient will mitigate the effects of whether to send gifts and gift types on the physician’s behavior. Our results suggest that a strong tie helps patients obtain high-quality medical services from physicians in an online health environment.

Based on the systematic empirical analyses on the motivation and effects of gift giving in an online health community, we have revealed the nature and mechanism of online gift giving. The value of the gift ranges from 5 to 200 RMB (approximately US $1 to $30), which is too small to be judged as “a red envelope” (cash wrapped in an envelope). Furthermore, an online virtual gift can improve the efficiency of physician-patient communication and contribute to the establishment of a good physician-patient relationship.

### Theoretical Implications

This study contributes to theory from four aspects. First, to the best of our knowledge, this study is among the first to explore the motivations and consequences of gift giving in online health communities. Although previous studies have questioned the value of gift giving in the health sector (mainly in hospital settings) and even treat gift giving as a reason to explain the current tense physician-patient relationship and moral reduction in the medical field [36,37], the specific factors that influence gift giving in an online health environment have been unknown. In contrast to the study of Zhao et al [14], both the details of reply and bedside manner of physicians were included as potential consequences of gift giving in our study. Therefore, we empirically confirmed the role of gift giving in an online health environment.

Second, this study contributes to gift theory and tie strength theory by empirically examining the different effects of gift giving on receiver behaviors in different giver-receiver relationships, which were measured by tie strength. By using unique interaction data between physicians and patients in an online forum, this study revealed significant moderating effects of tie strength on the relationships between gift giving and physicians’ behaviors. Our results suggest that environmental factors need to be carefully considered to make an accurate judgment on the effects of gift giving.

Third, to the best of our knowledge, this study is among the first to empirically explore the different effects of gift types, especially in health care. Based on the characteristics of both affective and instrumental gifts, we first coded each gift by analyzing the whole interaction process between physicians and patients and then examined their respective effects. These results contribute to the current literature on gift giving by demonstrating that these two types of gifts have different impacts on the improvement of physicians’ service quality.

Fourth, this study contributes to prior studies by using a large sample with a real operation date and analysis under strict criteria. Compared with survey data, our data were collected from the open transaction platform and are therefore more practical. Moreover, text mining was used to conduct sentiment analysis of the whole interaction content between physicians and patients. Text mining can help to obtain more comprehensive and accurate information with respect to an individual’s emotion.

### Practical Implications

The practice of gift giving has been questioned since it was first launched on online health communities, even though patients are not obligated to buy physicians gifts. Therefore, exploring the motivations and impacts of gift giving in medical services in online health communities is important because it can provide guidance on how to improve communication efficiency. All of our results suggest that giving online virtual gifts is of considerable significance to promoting effective physician-patient communication and is conducive to the relief of physician-patient conflicts. This study offers several important practical implications for both online health community users and administrators.

For patients, a gift can be provided to physicians when the patient desires better service, as we found that digital gifts may lead to a satisfactory reply during the interaction between patients and physicians. Moreover, patients should be aware that the gift will be more powerful when their relationship with the physician is more alienated. In addition, it is better to give an affective gift in the early stage of the interaction, as these types of gifts have a more powerful influence when the patient and the physician are strangers.

For administrators of online health communities, a more useful mechanism should be established to encourage patients to give digital gifts to the physician when necessary, such as construction of a more convenient interface. In addition, reminders can be set for patients to send a gift to physicians at the right time. Moreover, the site should not immoderately encourage patients to give gifts to physicians. The original objective of the gift-giving function is to provide a channel for patients to express their appreciation to physicians. Excessive gift giving will obstruct the development of good relations between physicians and patients. Therefore, we suggest that the administrators of online health communities set a suitable mechanism to moderate guidance for gift giving.

### Limitations and Future Research

The limitations of this study can offer potential directions for future research. First, we did not obtain information on patients such as age, gender, and occupation, as patient information is rarely disclosed on the online health communities in China for privacy protection. Future research can attempt to obtain more thorough data or to eliminate the influences of patient characteristics using more complex techniques or methods. Second, as the number of physician replies by voice messages in the physician-patient interact process was very small, we deleted these from our analysis. However, voice messages may also include relevant information and may be more important than text messages as voice can convey emotion. Future studies should therefore attempt to obtain more data on voice content and useful related information.

## Appendix

Multimedia Appendix 1 Chinese terms expressing demand.

Multimedia Appendix 2 Descriptive statistics and correlations among variables.

Multimedia Appendix 3 Extraction of feature words from physician-patient dialogs.
