# Supplementary material for: Consequences of Gift Giving in Online Health Communities on Physician Service Quality: Empirical Text Mining Study
Source: J Med Internet Res. 2020 Jul 30;22(7):e18569. doi: 10.2196/18569 (PMC7426794; doi:10.2196/18569)
Supplement: Multimedia Appendix 1 [file jmir_v22i7e18569_app1.docx]

**Multimedia Appendix 1:** Chinese terms expressing demand

| Vocabulary | | | | | |
| --- | --- | --- | --- | --- | --- |
| Why:  为什么;  为啥 | How:  怎么;  如何 | What:  什么 | Which:  哪些;  哪种 | How long:  多久 | Where:  哪里 |
| Could:  行吗;  好吗;  能不能;  会不会;  是吧 | But:  但是;  可;  不过 | How many:  多少;  几 | Confusion:  吗;  呢;  ？;  有问题;  不知道;  不舒服 | If:  是吗;  是否;  需不需要;  可不可以;  要不要;  是不是 | Emotion:  拜托;  请;  打扰;  想;  帮;  问;  求;  请教;  麻烦;  不好意思;要紧;  咨询 |
