# Supplementary material for: Consequences of Gift Giving in Online Health Communities on Physician Service Quality: Empirical Text Mining Study
Source: J Med Internet Res. 2020 Jul 30;22(7):e18569. doi: 10.2196/18569 (PMC7426794; doi:10.2196/18569)
Supplement: Multimedia Appendix 2 [file jmir_v22i7e18569_app2.docx]

Multimedia Appendix 2. Descriptive statistics and correlations among variables.

| Variable | Mean (SD) | *Ratio_WordCount_ij_* | *PhonePrice_j_* | *WrittenPrice_j_* | *Outpatient_j_* | *Thank-you Letter_j_* | *Contribution_j_* | *No. Gifts_j_* | *No. Patients_j_* | *Recommendation_j_* | *Title_j_* | *City_j_* | *Level_j_* | *GiftGiving_ij_* | *GiftType_ij_* |
| --- | --- | --- | --- | --- | --- | --- | --- | --- | --- | --- | --- | --- | --- | --- | --- |
| *Ratio_WordCount_ij_* | 1.276 (1.156) |  |  |  |  |  |  |  |  |  |  |  |  |  |  |
| *PhonePrice_j_* | 4.509 (0.753) | –0.045^**^ |  |  |  |  |  |  |  |  |  |  |  |  |  |
| *WrittenPrice_j_* | 4.381 (0.828) | –0.013^*^ | 0.788^**^ |  |  |  |  |  |  |  |  |  |  |  |  |
| *Outpatient_j_* | 0.360 (0.479) | 0.017^**^ | 0.272^**^ | 0.259^**^ |  |  |  |  |  |  |  |  |  |  |  |
| *Thank-you Letter_j_* | 3.702 (0.964) | 0.031^**^ | 0.451^**^ | 0.573^**^ | 0.261^**^ |  |  |  |  |  |  |  |  |  |  |
| *Contribution_j_* | 10.18 (1.042) | 0.043^**^ | 0.230^**^ | 0.398^**^ | 0.243^**^ | 0.766^**^ |  |  |  |  |  |  |  |  |  |
| *No. Gifts_j_* | 5.367 (1.186) | 0.020^**^ | 0.328^**^ | 0.485^**^ | 0.177^**^ | 0.822^**^ | 0.873^**^ |  |  |  |  |  |  |  |  |
| *No. Patients_j_* | 7.580 (0.992) | 0.049^**^ | 0.281^**^ | 0.429^**^ | 0.318^**^ | 0.797^**^ | 0.952^**^ | 0.823^**^ |  |  |  |  |  |  |  |
| *Recommendation_j_* | 4.429 (0.296) | 0.006 | 0.429^**^ | 0.517^**^ | 0.246^**^ | 0.731^**^ | 0.643^**^ | 0.702^**^ | 0.621^**^ |  |  |  |  |  |  |
| *Title_j_* | 0.700 (0.459) | 0.025^**^ | 0.124^**^ | 0.113^**^ | 0.285^**^ | 0.286^**^ | 0.281^**^ | 0.268^**^ | 0.340^**^ | 0.233^**^ |  |  |  |  |  |
| *City_j_* | 0.200 (0.403) | –0.019^**^ | 0.224^**^ | 0.180^**^ | –0.034^**^ | –0.109^**^ | –0.185^**^ | –0.137^**^ | -0.202^**^ | –0.092^**^ | –0.136^**^ |  |  |  |  |
| *Level_j_* | 0.910 (0.293) | –0.038^**^ | 0.075^**^ | 0.052^**^ | 0.241^**^ | 0.095^**^ | 0.093^**^ | 0.092^**^ | 0.099^**^ | 0.073^**^ | 0.087^**^ | –0.002 |  |  |  |
| *GiftGiving_ij_* | 0.650 (0.478) | 0.078^**^ | 0.016^*^ | 0.029^**^ | 0.003 | 0.035^**^ | 0.016^**^ | 0.042^**^ | 0.024^**^ | 0.022^**^ | 0.007 | 0.001 | –0.008 |  |  |
| *GiftType_ij_* | 0.180 (0.387) | –0.010 | 0.030^**^ | 0.011 | 0.030^**^ | –0.008 | 0.008 | –0.025^**^ | 0.016^**^ | –0.011 | 0.016^**^ | –0.003 | 0.002 | 0.349^**^ |  |
| *TieStrength_ij_* | 0.000 (1.731) | –0.059^**^ | –0.059^**^ | –0.043^**^ | –0.078^**^ | –0.062^**^ | –0.012^*^ | 0.016^**^ | –0.054^**^ | –0.012^*^ | –0.040^**^ | 0.020^**^ | 0.030^**^ | 0.117^**^ | –0.079^**^ |

****P*<.001; ***P*<.01; **P*<.05.
