# Supplementary material for: Consequences of Gift Giving in Online Health Communities on Physician Service Quality: Empirical Text Mining Study
Source: J Med Internet Res. 2020 Jul 30;22(7):e18569. doi: 10.2196/18569 (PMC7426794; doi:10.2196/18569)
Supplement: Multimedia Appendix 3 [file jmir_v22i7e18569_app3.docx]

**Multimedia Appendix 3:**

We imported the dialogues between physicians and patients into Microsoft Excel, as shown in the first screenshot. This text content was then input to TextMind; part of the output of the processing is shown in the screenshot below. A total of 102 feature words were obtained through TextMind software. The feature word “Positive Emotion” (in bold) represents positive emotion of the dialogue, and the value in the column represents the positive emotional tendency.


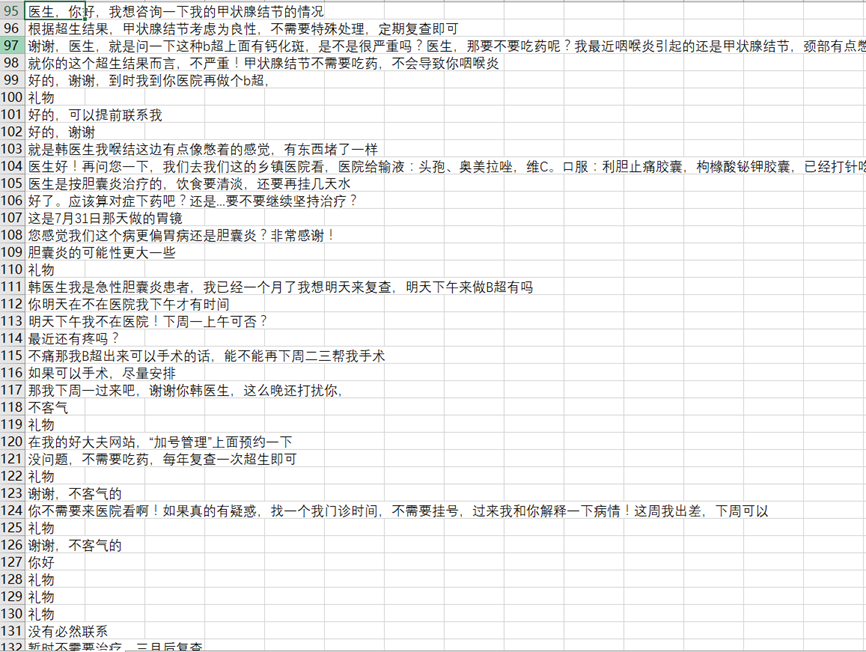


Input context


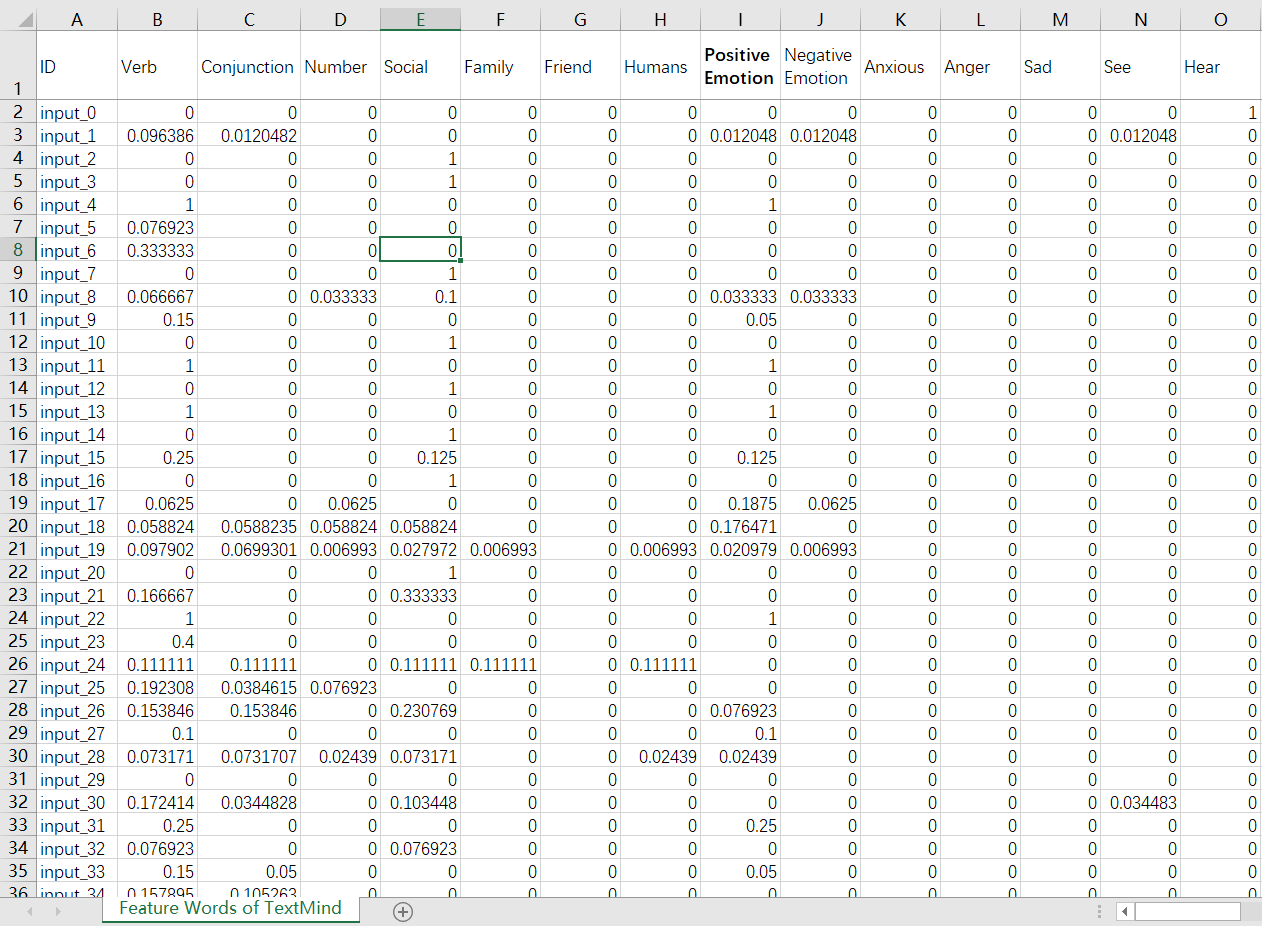


Output results
